# A Closed-Loop Active Vision and Reinforcement Learning Framework for Online Optical Lens Inspection in Computer-Integrated Manufacturing

**DOI:** 10.3390/s26216878

**Published:** 2026-10-30

**Authors:** Yikai Yang, Xuedong Jing

**Affiliations:** Faculty of Intelligence Technology, Shanghai Institute of Technology, Shanghai 201418, China; lyyk0519@gmail.com

**Keywords:** optical lens defect detection, machine vision, active vision, reinforcement learning, computer-integrated manufacturing

## Abstract

Automated defect inspection for transparent optical lenses remains challenging because specular reflections can obscure weak defects and produce glare-induced false responses. This paper presents a confidence-driven end-to-end active inspection framework for online quality control in computer-integrated manufacturing. The framework integrates a Transformer-enhanced You Only Look Once (YOLO) detector (TE-YOLO), a confidence-driven proximal policy optimization (PPO) controller (CDR-PPO), and a time-sequential camera viewpoint adjustment process. TE-YOLO introduces a C3 module with a Transformer encoder (C3TR) before the detection head to combine convolutional local feature extraction with global context modeling for scratch and foreign particle detection. In the proposed architecture, the detector outputs both visual features and defect confidence scores, and these outputs are converted into a threshold-aware reward signal that directly participates in PPO advantage estimation and policy optimization, thereby coupling visual perception with discrete camera pose control. Unlike active vision methods whose rewards are based on geometric coverage or model uncertainty, CDR-PPO trains the viewpoint policy against the same detector threshold that accepts an observation. The resulting closed loop repeatedly performs image acquisition, defect perception, reward construction, policy update, camera adjustment, and re-observation; the detector and the policy are trained separately. The lens images are acquired on a physical inspection apparatus with an industrial camera on an adjustable support. TE-YOLO is evaluated on 208 physical lens images, where it reaches an average precision at an intersection-over-union threshold of 0.50 (AP50) of 0.995 and an average precision averaged over thresholds from 0.50 to 0.95 (AP50:95) of 0.865 on a near-duplicate-grouped split, the same accuracy as that of a plain C3 variant without the Transformer encoder. Physical acquisition sequences on the apparatus show that changing the camera configuration shifts the glare relative to the lens and the defect. CDR-PPO is trained and evaluated in a simulated inspection environment that reproduces the geometry of the apparatus and generates the confidence and the state features. On held-out test scenarios, it reaches the confidence threshold in 87.3% of episodes with 5.1 observations per episode, compared with 42.4% for a fixed viewpoint and 22.1 observations per episode for a greedy confidence search. Within a budget of 10 observations, CDR-PPO recovers 77.8% of the episodes that start below the threshold, whereas the greedy search recovers none. These results support the integration of adaptive visual inspection into automated manufacturing quality control workflows.

## 1. Introduction

Optical lenses are fundamental components in modern precision imaging systems. However, the manufacturing process inevitably introduces surface and internal defects. Strict quality control is imperative, yet manual inspection is difficult to scale and is susceptible to operator variability and fatigue [1]. Consequently, automated machine vision systems, particularly one-stage detectors like the You Only Look Once (YOLO) family, have been widely adopted for real-time surface defect inspection [2,3].

Despite the success of standard convolutional neural networks (CNNs), transparent and highly reflective optical lenses pose severe challenges. Existing passive automated solutions face two fundamental limitations. First, defects on optical lenses are typically characterized as “weak micro-defects” with an ambiguous morphology, low contrast, and small spatial extent [2,4,5,6]. Although deep CNNs can obtain large theoretical receptive fields, their effective feature aggregation remains locally biased, which may limit long-range modeling on highly reflective metal or glass surfaces [7]. While effective for extracting prominent textures, they often misclassify high-frequency reflection noise as microscopic defects due to the lack of global semantic understanding of the complex lighting environment.

Second, a fixed camera pose cannot adaptively avoid glare areas [8,9,10]. A recent comprehensive review indicates that while machine learning vision systems achieve high accuracy in controlled environments, over 77% remain at the prototype scale, facing systematic deployment barriers in complex physical setups [11]. When a defect overlaps with a severe specular highlight, the visual information is physically lost at the sensor level, substantially limiting the information available to any subsequent image processing algorithm.

To overcome these coupled challenges, we adopt an active vision paradigm [9]. Active vision refers to a perception strategy in which the sensing system actively changes its viewpoint or acquisition configuration to obtain more informative observations, rather than processing images captured from a fixed pose. In the proposed framework, this principle is implemented by coupling global context defect perception with iterative camera pose adjustment.

In this work, we propose a unified end-to-end framework integrating visual perception, confidence-driven policy learning, and time-sequential camera control. The term end-to-end refers to the complete closed-loop processing chain from raw image acquisition and defect perception to threshold-aware reward construction, proximal policy optimization (PPO), camera pose adjustment, and subsequent re-observation. It does not imply joint optimization through a shared back-propagation path: the detector is trained separately, and its outputs serve as policy inputs and reward signals. Specifically, Transformer-enhanced YOLO (**TE-YOLO**) addresses the perception challenge by introducing global context modeling before the detection head, whereas confidence-driven PPO (**CDR-PPO**) addresses the physical acquisition challenge by converting detector outputs into a reward signal that guides discrete camera position and orientation adjustments. The dynamic observation sequence explicitly represents how the sensing state evolves at successive decision intervals.

Existing optical lens inspection methods improve the detector applied to images acquired from a fixed camera and illumination setup, whereas active vision and reinforcement learning inspection methods move the sensor but typically reward geometric coverage or reductions in model uncertainty. The proposed framework couples the two: the viewpoint policy is trained on the post-action detector confidence and on the same threshold that accepts an inspection. In this study, all lens images are acquired on a physical inspection apparatus. TE-YOLO is trained and tested on these images, and physical acquisition sequences on the same apparatus show how changes in the camera configuration move the glare on the lens. CDR-PPO is trained and compared with baseline policies in an inspection environment that reproduces the apparatus geometry, which allows a paired and repeatable evaluation over thousands of episodes. In this environment, the confidence and the state features are generated by a model of the relation between the camera pose, glare, and defect visibility.

The main contributions of this work are summarized as follows:

1. We develop **TE-YOLO**, in which a C3 module with a Transformer encoder (C3TR) is placed before the detection head to complement YOLO-based local feature extraction with global context reasoning for glare-affected scratch and foreign particle detection, and we evaluate the module through a seed-paired ablation against a plain C3 variant together with its computational cost.
2. We develop **CDR-PPO**, a PPO-based active view controller whose task-specific reward is constructed from the defect confidence, a confidence threshold bonus and camera motion cost. The thresholded detector response is therefore used directly as a policy training signal. We derive the condition on the reward weights under which reaching the threshold is preferred to remaining just below it, and a controlled ablation shows that the threshold bonus, weighted according to this condition, is the essential component of the reward.
3. We establish a **dynamic observation protocol** that represents the iterative sequence of image acquisition, defect inference, policy decision, camera pose adjustment, and re-observation over time, thereby linking the perception and control modules within a unified inspection space.

## 2. Related Works

### 2.1. YOLO-Based Optical Defect Detection and Global Context Modeling

Recent lens inspection studies have evaluated YOLO-based detectors on dedicated optical lens datasets, while widely used industrial benchmarks such as MVTec AD have supported the broader development of data-driven anomaly and defect inspection [12,13]. Historically, the inspection of optical lenses has heavily relied on manual visual checks by human experts. However, manual inspection is inherently subjective, prone to fatigue, and suffers from low accuracy and limited scalability [1]. Early automated attempts employed traditional machine vision techniques, utilizing handcrafted feature extractors such as Sobel edge detection, thresholding, and morphological operations. While these methods provided a degree of automation, they proved highly sensitive to illumination variations and were largely incapable of detecting low-contrast and complex morphological defects [4].

The introduction of deep learning, particularly convolutional neural networks (CNNs), substantially improved automated surface defect inspection [1]. Two-stage detectors like Faster R-CNN improved the localization accuracy, but their computational overhead hindered real-time deployment. Consequently, one-stage detectors, particularly the YOLO family, have been widely adopted because of their favorable balance between detection accuracy and inference efficiency [3,4].

Recent advancements have extensively deployed YOLO variants for high-speed industrial detection. Researchers have introduced multi-scale feature fusion (e.g., spatial pyramid pooling-fast (SPPF) modules) and lightweight network structures to tackle the challenges of “weak micro-defects” and low-contrast targets commonly found on lens surfaces [2,3,4]. Furthermore, techniques such as anchor optimization and ghost modules have been utilized to refine the detection of irregular surface anomalies [14].

Despite these significant achievements, purely CNN-based architectures remain biased toward local feature aggregation. While convolutions are highly effective at extracting prominent local textures, they lack a holistic understanding of the global lighting environment [7]. For transparent and highly reflective materials like optical lenses, this local bias makes CNNs highly susceptible to false positives, frequently misclassifying high-frequency specular reflections (glare) as structural defects.

### 2.2. Transformer-Enhanced Detection for Reflective Surfaces

To complement the locally biased feature aggregation of CNNs, the computer vision community has increasingly turned to attention mechanisms and Vision Transformers. This paradigm shift was largely catalyzed by the introduction of DETR (Detection Transformer) [15], which successfully reframed object detection as a direct set prediction problem. By employing a bipartite matching loss (via the Hungarian algorithm) and a multi-head self-attention mechanism, DETR introduced global self-attention into object detection and formulated detection as direct set prediction, thereby avoiding hand-designed anchor boxes and non-maximum suppression (NMS).

In the specific domain of industrial defect detection, Transformer-based models have shown promising performance in handling complex and ambiguous defect morphologies. For instance, STMask R-CNN successfully incorporated the Swin Transformer to enhance the modeling capabilities for lens defects in complex environments [16]. However, pure Transformer architectures can incur substantial computational costs, making them challenging to deploy in real-time manufacturing pipelines [17,18,19].

To balance the global reasoning of Transformers and the real-time efficiency of CNNs, recent approaches have proposed hierarchical and deformable attention designs [19,20]. Architectures integrating deformable attention, dynamic spatial modeling, and triplet attention—such as TAFF-YOLO [21], MDEB-YOLO [22], and YOLO-DST [23]—have been utilized to detect micro-defects on printed circuit boards and semiconductor chips. Real-time end-to-end detectors further demonstrate that Transformer-based detection can be designed for efficient inference [24]. A common consensus among these advanced methods is that global self-attention is highly effective at enhancing critical defect features while explicitly suppressing irrelevant background noise and texture interference. Inspired by these structural innovations, these studies motivate the use of a C3TR module in the proposed TE-YOLO perception branch. In our architecture, the module is placed before the detection head so that the final prediction stage receives both locally encoded features and global context information. The intended role is to improve discrimination between localized defects and spatially distributed reflection patterns; the module itself is not presented as a newly invented Transformer architecture.

### 2.3. Reinforcement Learning and Time-Sequential Active Observation

While algorithmic improvements in detection networks have significantly advanced the field, passive vision systems remain physically constrained by fixed camera viewpoints. A recent comprehensive review highlights a critical bottleneck: although machine learning vision systems can achieve near-perfect accuracy in controlled laboratory environments, over 77% of these implementations remain stuck at the prototype scale, facing systematic deployment barriers in complex, unstructured physical setups [11]. When a physical defect is completely obscured by a severe specular highlight from a specific viewing angle, the visual information is lost at the sensor level, rendering any algorithmic post-processing ineffective.

Active vision addresses this fundamental physical limitation by enabling embodied agents to dynamically control their sensory inputs to improve perception, shifting the paradigm from passive observation to autonomous, goal-directed exploration [8,9,25]. Reinforcement learning (RL) has emerged as a natural and powerful mathematical framework for this sequential decision-making process. Recent reviews on AI-empowered industrial robotics emphasize that deep reinforcement learning (DRL) is increasingly employed to learn adaptive strategies rather than relying solely on hard-coded rules [26,27].

Recent robotic inspection and next-best-view systems formulate active perception as sequential decision-making under partial observability [10,28]. For example, uncertainty-aware active inspection uses deep reinforcement learning to guide viewpoint selection based on the segmentation uncertainty [28]. By calculating the pixel-wise uncertainty of predictions across multiple time steps, the agent is driven to viewpoints that actively reduce this uncertainty.

However, directly applying complex uncertainty estimation networks to real-time optical lens inspection entails significant computational overhead and latency, which is detrimental to high-throughput manufacturing. Furthermore, standard RL algorithms often struggle with sparse rewards in sequential viewpoint control tasks, requiring careful reward shaping to guide the agent efficiently [29,30]. To bridge this gap, and drawing inspiration from recent works on intrinsic sensorimotor reward designs [31], this line of research motivates the proposed CDR-PPO controller. Rather than modifying the core PPO optimization rule, CDR-PPO introduces a task-specific confidence-driven reward and couples this reward to the detector output. The contribution therefore lies in reward construction, perception–control coupling, and its application to sequential lens viewpoint adjustment, rather than in claiming a new generic policy gradient algorithm.

Reward shaping adds auxiliary terms to a sparse task reward to accelerate learning, and potential-based shaping, $F\left(s,s^{\prime }\right)=\gamma \Phi \left(s^{\prime }\right)-\Phi \left(s\right)$, leaves the set of optimal policies unchanged [29]. The confidence-driven reward (CDR) differs in two respects. First, its shaping signal is the post-action response of the detector that also makes the inspection decision, so the policy is trained on the same quantity and against the same threshold that determines whether an observation is accepted. Second, it adds the absolute confidence at every step rather than a potential difference. This yields a dense signal but can change the optimal policy, which motivates the weight condition derived in Section 3.2.2 and the comparison with a potential-based variant in Section 4.3.2.

## 3. Proposed Approach

**Figure 1:**
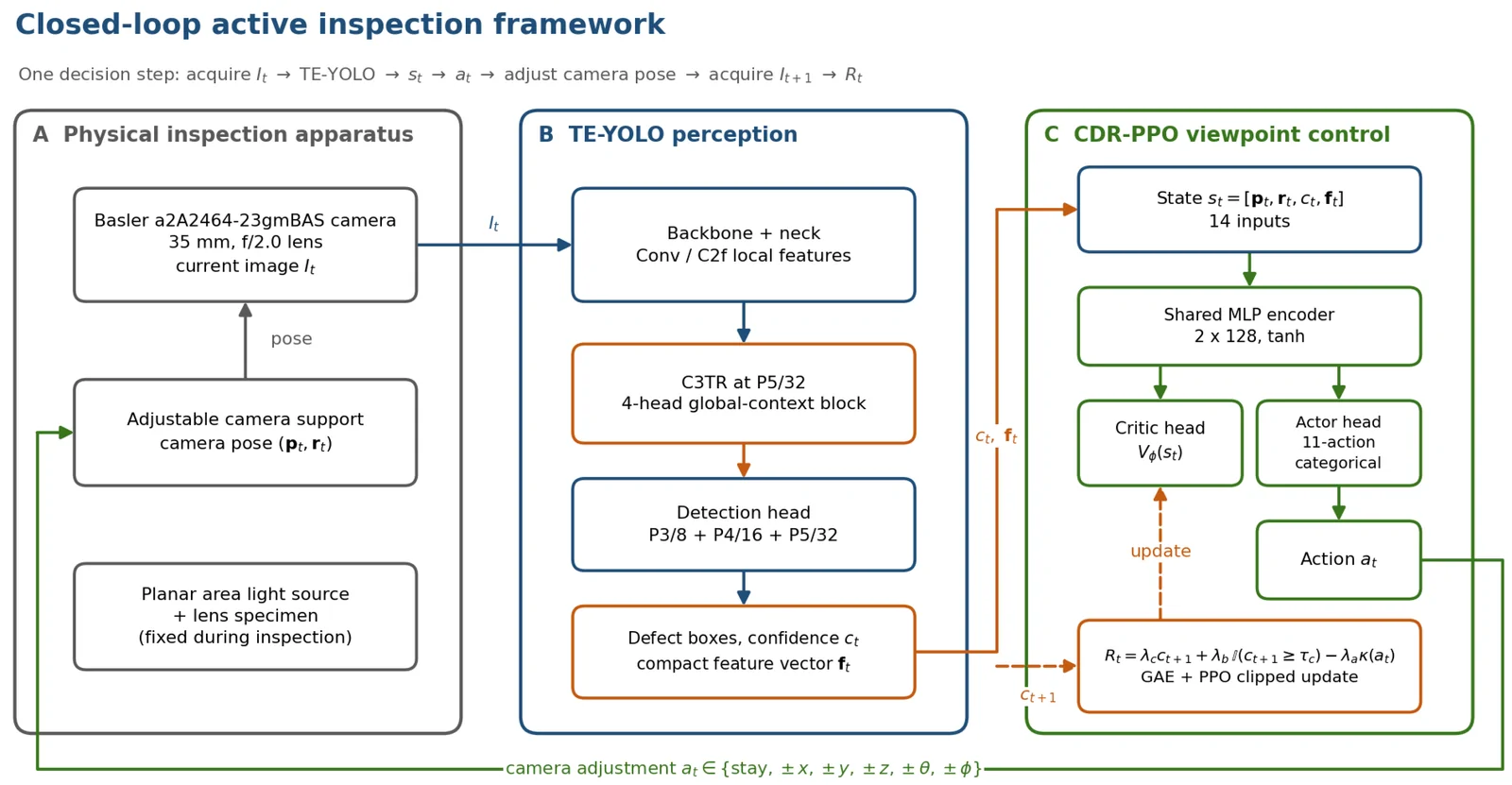
Closed-loop active inspection framework. (**A**) The physical inspection apparatus acquires the lens image $I_{t}$ at the current camera pose. (**B**) TE-YOLO converts the image into defect predictions, the confidence $c_{t}$, and the compact feature vector $f_{t}$. (**C**) CDR-PPO maps the state $s_{t}=\left[p_{t},r_{t},c_{t},f_{t}\right]$ to one of 11 discrete camera adjustments and is trained with the confidence-driven reward computed from the post-action confidence $c_{t+1}$. The figure shows the proposed architecture; in the policy experiments of this work, $c_{t}$ and $f_{t}$ are generated by the inspection environment (Section 4.1). Arrows show the sequence from image acquisition to the next camera adjustment, and bold symbols denote vectors.

Figure 1 summarizes the proposed framework. In this section, we detail the proposed end-to-end active vision framework, which comprises the **TE-YOLO** perception module, the **CDR-PPO** decision module, and a dynamic observation process that iteratively updates the camera viewpoint over time. Here, “end-to-end” denotes the complete closed-loop processing chain from image acquisition and defect perception to reward construction, viewpoint decision, camera adjustment, and subsequent observation. It does not imply that the detector and policy network are optimized through a single shared back-propagation path.

### 3.1. TE-YOLO: Global Context Perception

Standard YOLO models excel in real-time inference but struggle with global context modeling, making them susceptible to high-frequency specular reflections on optical lenses. To address this, we propose **TE-YOLO** by inserting a **C3TR module** before the detection head. C3TR is a hybrid C3-based module that incorporates a Transformer encoder; accordingly, the redundant expression “C3TR Transformer” is avoided because the suffix “TR” already denotes the Transformer component.

#### Architecture Design

**Figure 2:**
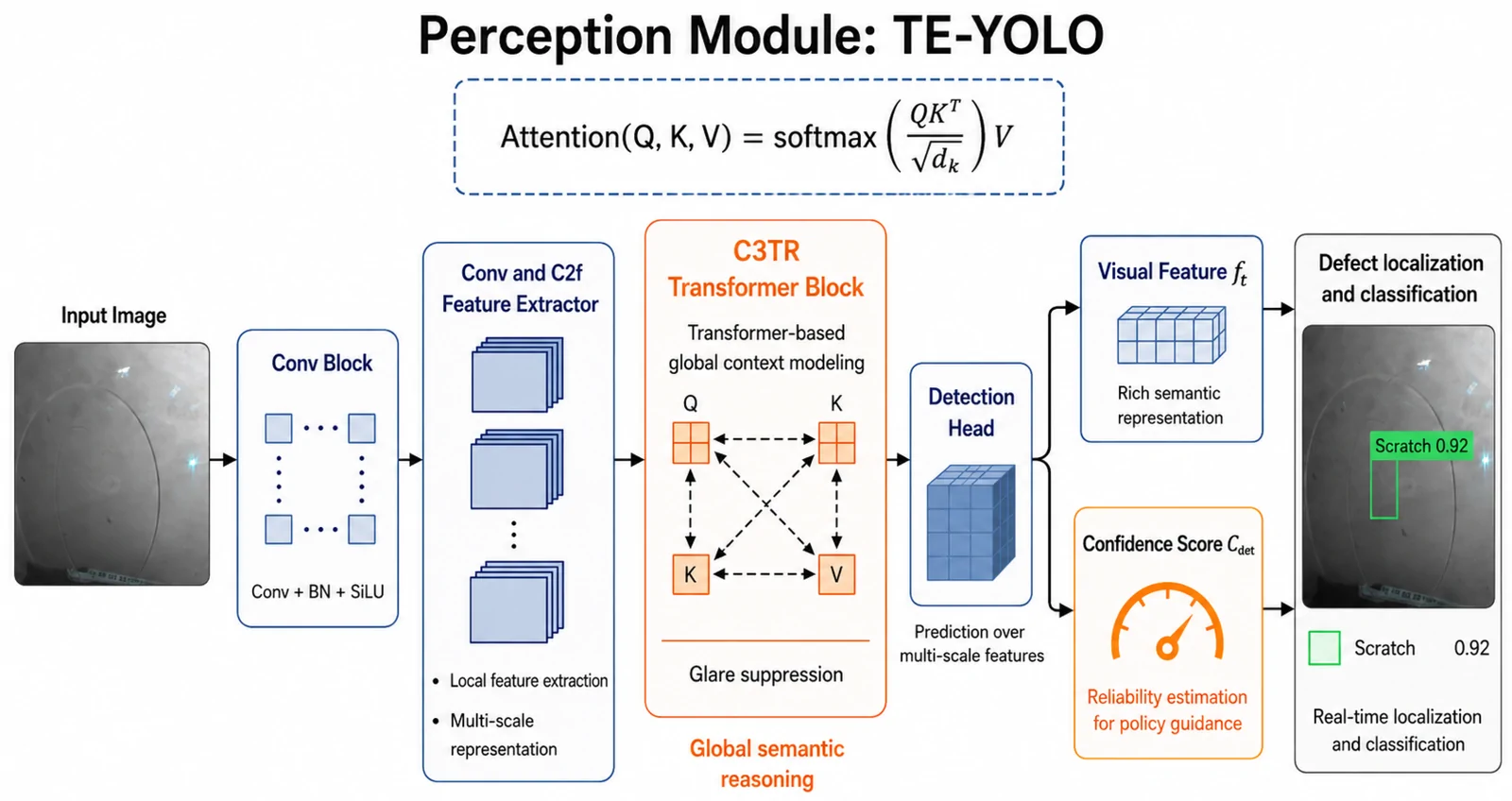
Detailed architecture of the TE-YOLO perception module. The Conv/C2f backbone extracts local visual textures, while the C3TR module performs global context modeling, and the detector outputs both final predictions and the intermediate signals that the proposed architecture reuses for active control, including the compact visual feature $f_{t}$ and the defect-oriented confidence branch. Ellipses denote repeated blocks of the same type. Blue marks the convolutional backbone, and orange marks the C3TR module and the confidence used for control.

TE-YOLO uses the YOLOv8n backbone (Conv, C2f, and SPPF blocks) and a path aggregation network (PAN) neck with a single DEFECT output class. In the last neck stage, which produces the stride-32 feature map passed to the detection head, the C2f block is replaced with a C3TR block containing a four-head Transformer encoder; all other layers are unchanged. This module employs Multi-Head Self-Attention (MHSA) to capture long-range dependencies across the spatial domain [15]. For an input feature map flattened into a sequence of queries (*Q*), keys (*K*), and values (*V*), the attention is computed as

(1) $$\mathrm{Attention}\left(Q,K,V\right)=\mathrm{softmax}\left(\frac{QK^{T}}{\sqrt{d_{k}}}\right)V$$

where $d_{k}$ denotes the dimensionality of the key vectors. The resulting global context representation is intended to support the distinction between localized defect patterns and spatially distributed specular reflections. Figure 2 further details the perception branch: the glare-affected raw lens image is first encoded by the Conv/C2f backbone for local texture extraction; it is then processed by the C3TR module for global context reasoning; and it is finally mapped to both the detection output and the reusable intermediate signals, namely the compact visual feature $f_{t}$ and the defect-oriented confidence score.

### 3.2. CDR-PPO: Active Viewpoint Planning

To overcome the physical limitations of fixed-angle inspection, we formulate active viewpoint selection as a discrete-time Markov Decision Process (MDP) defined by a tuple 〈S,A,R,P,γ〉. Each decision step corresponds to one camera adjustment cycle. A nominal interval of 30 s is assigned to each cycle in the policy experiments; on the apparatus, the interval is determined by camera motion, settling, and image acquisition and can be adapted to the production takt.

#### 3.2.1. State and Action Space

To enrich the reinforcement learning state space and capture the physical interaction between the camera pose, glare, and defect visibility, the state $s_{t}\in S$ at time step *t* is defined as

(2) $$s_{t}=\left[p_{t},r_{t},c_{t},f_{t}\right],$$

where $p_{t}={\left[x_{t},y_{t},z_{t}\right]}^{T}$ denotes the camera position, $r_{t}={\left[\theta_{t},\phi_{t}\right]}^{T}$ denotes pitch and yaw, $c_{t}$ is the defect-oriented detection confidence, and $f_{t}$ is the compact visual feature vector extracted by TE-YOLO. This definition describes the proposed architecture. In the policy experiments reported in this paper, $c_{t}$ is a temperature-calibrated confidence and $f_{t}$ is an eight-dimensional feature vector, both generated by the inspection environment (Section 4.1), so that the policy has 14 inputs. On real images, the corresponding quantities would be obtained as follows: $c_{t}$ is the highest TE-YOLO box confidence, temperature-calibrated on held-out images; the pose is read from the camera support; the illumination intensity is taken from the light source setting or from the mean image brightness; glare features are computed from the area, centroid, and intensity gradient of the saturated pixels; and the view quality can be approximated by an image sharpness measure. The three offsets to the preferred pose and the surface roughness in $f_{t}$ are defined by the environment, so that, for operation on the apparatus, the state is restricted to the observable quantities above. The control process is discretized in time, with one decision produced per camera adjustment cycle. In the present implementation, the admissible camera adjustments are further represented by the following discrete action set:

(3) $$a_{t}\in A=\left\{\mathrm{stay},\pm x,\pm y,\pm z,\pm \theta ,\pm \phi \right\}.$$

Each action denotes one fixed-step displacement or angular adjustment of the camera. The step size is 0.16 in normalized pose coordinates and is held constant in all policy comparisons; the lens, the light source, and the receiving screen remain fixed, and exposure is not controlled. The detector reports one confidence value per predicted defect box. For policy learning, $c_{t}$ denotes the confidence associated with the highest-confidence defect prediction in the current observation. For a normal lens, an inspection is correct when no defect prediction exceeds the operating threshold over the observation sequence. The glare ratio $g_{t}$ is computed by the inspection environment and is used only as an evaluation metric; it is not a policy input, a reward term, or a termination condition. The detector confidence provides the direct visual feedback for policy learning: viewpoints that improve defect visibility increase $c_{t}$, whereas glare-obscured observations yield lower confidence. Table 1 lists these definitions.

#### 3.2.2. Confidence-Driven Dense Reward

The main design of the CDR-PPO controller is the confidence-driven reward (**CDR**) function. Instead of relying only on a sparse terminal reward, we combine the continuous defect confidence, a threshold bonus, and camera motion economy:

(4) $$b_{t+1}=I\left(c_{t+1}\geq \tau_{c}\right),$$

(5) $$R_{t}=\lambda_{c}c_{t+1}+\lambda_{b}b_{t+1}-\lambda_{a}\kappa \left(a_{t}\right),$$

Here, $b_{t+1}$ converts the confidence threshold decision into an explicit reward component, which is the central connection between TE-YOLO and PPO training. The reward $R_{t}$ is assigned to the transition $\left(s_{t},a_{t},s_{t+1}\right)$; its confidence and threshold terms use the observation acquired after $a_{t}$, and $\kappa (a_{t})$ assigns a predefined cost to the executed translation or rotation action. The continuous term $c_{t+1}$ provides dense visual guidance, and $b_{t+1}$ supplies an additional bonus after the detector confidence exceeds $\tau_{c}$. The reward is accumulated into the return and advantage estimates used by PPO. Consequently, the detector threshold affects the policy update rather than serving only as a post-processing rule. Threshold attainment is declared when $c_{t+1}\geq \tau_{c}$, and the episode then terminates.

*Weight condition*. Because the confidence term is paid at every non-terminal step, a policy that keeps $c_{t+1}$ just below $\tau_{c}$ collects a discounted return approaching $\lambda_{c}\tau_{c}/\left(1-\gamma \right)$, whereas reaching the threshold yields at least $\lambda_{c}\tau_{c}+\lambda_{b}$ and ends the episode. Neglecting the motion cost, reaching the threshold is therefore preferred to remaining just below it only if

(6) $$\lambda_{b}>\frac{\lambda_{c}\tau_{c}\gamma }{1-\gamma }.$$

With $\tau_{c}=0.75$ and $\lambda_{c}=1$, the bound is 74.25 for γ=0.99, 14.25 for γ=0.95, and 6.75 for γ=0.9. The threshold $\tau_{c}=0.75$ and the weights $\lambda_{c}=1$ and $\lambda_{a}=0.025$ are fixed in advance, and $\lambda_{b}$ and γ are selected on validation scenarios under this condition (Section 4.3.2).

*Scope of the condition*. Equation (6) assumes an unbounded accumulation of below-threshold rewards and neglects the motion cost. With *H* remaining steps, remaining just below the threshold yields at most $\lambda_{c}\tau_{c}\sum_{k=0}^{H-1}\gamma^{k}$, so reaching the threshold is preferred if $\lambda_{b}>\lambda_{c}\tau_{c}\gamma \left(1-\gamma^{H-1}\right)/\left(1-\gamma \right)$. This bound increases with *H* and remains below that of Equation (6); for H=20 and γ=0.9, it is 5.84 instead of 6.75. Equation (6) is therefore a sufficient condition for the 20-step episodes used here. The stay action has no motion cost, whereas reaching the threshold requires at least one camera move, which costs at most $\lambda_{a}$. Including this cost adds $\lambda_{a}$ to the right-hand side of Equation (6), which gives 6.775 for γ=0.9 and $\lambda_{a}=0.025$. The selected weight $\lambda_{b}=10$ satisfies all three bounds.

#### 3.2.3. Policy Optimization

The reward sequence is converted into advantage estimates with generalized advantage estimation (GAE), $\lambda_{\mathrm{GAE}}=0.95$. Let $d_{t}$ indicate a terminal transition after action $a_{t}$, and let $u_{t}$ indicate a truncation at the maximum number of adjustment steps. The temporal-difference residual, the advantage recursion, and the value target are

(7) $$\delta_{t}=R_{t}+\gamma \left(1-d_{t}\right)V_{\phi }\left(s_{t+1}\right)-V_{\phi }\left(s_{t}\right),$$

(8) $${\hat{A}}_{t}=\delta_{t}+\gamma \lambda_{\mathrm{GAE}}\left(1-d_{t}\right)\left(1-u_{t}\right){\hat{A}}_{t+1},\;{\hat{G}}_{t}={\hat{A}}_{t}+V_{\phi }\left(s_{t}\right).$$

For a terminal transition, the next-state value is masked out; for a time-limit truncation, the final observed state supplies the bootstrap value in $\delta_{t}$, and the recursion does not cross into the next episode. These advantages establish the explicit training path

(9) $$I_{t+1}\to c_{t+1}\to b_{t+1}\to R_{t}\to {\hat{A}}_{t}\to \pi_{\theta }\left(a_{t}|s_{t}\right).$$

**Figure 3:**
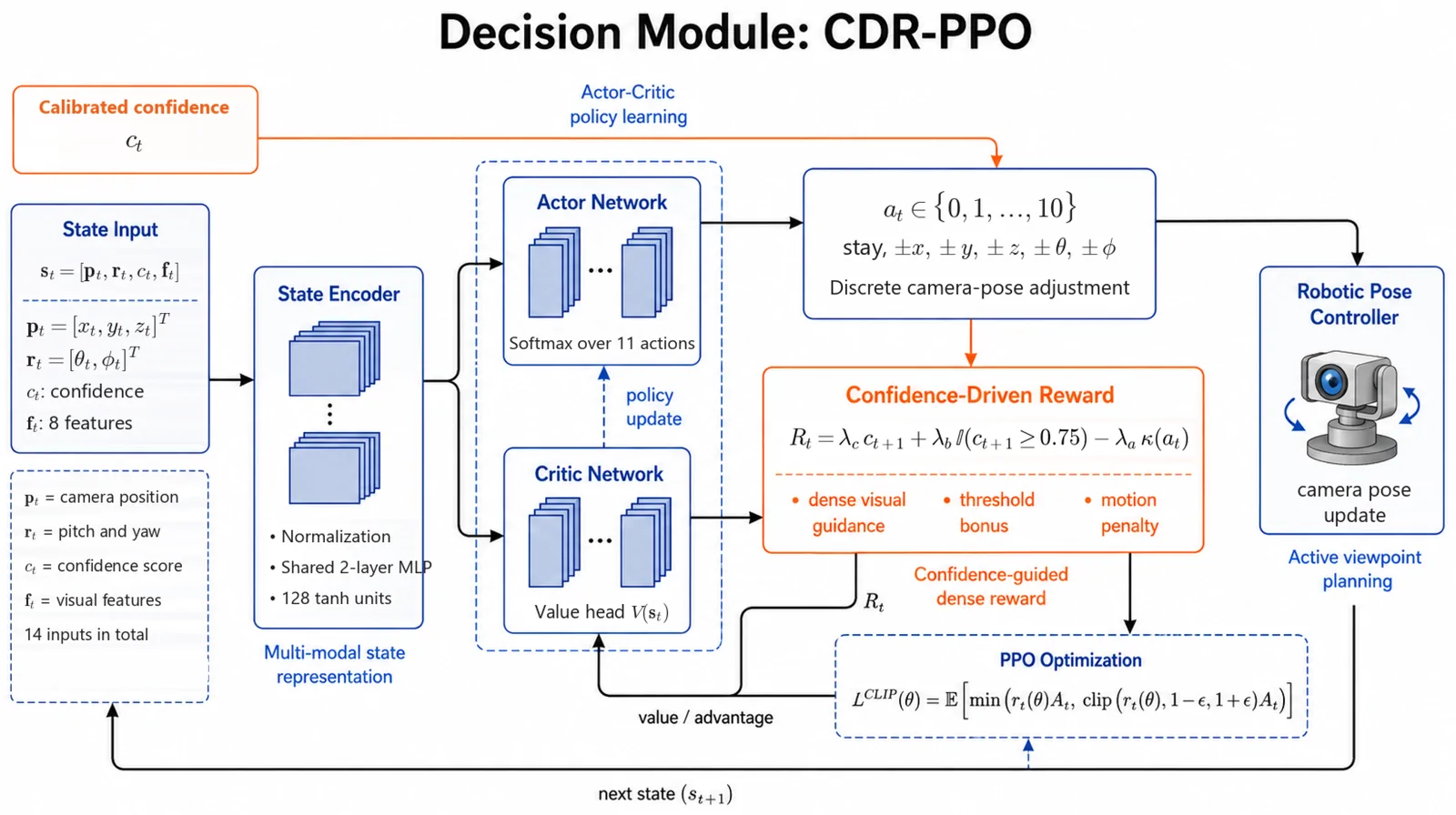
Detailed structure of the CDR-PPO decision module. The actor–critic policy consumes the multi-modal state $s_{t}=\left[p_{t},r_{t},c_{t},f_{t}\right]$ (14 inputs), selects one of 11 discrete camera pose actions, and is trained with the confidence-driven reward, which combines the post-action defect confidence, a threshold bonus, and a motion penalty. Ellipses abbreviate repeated layers of the same type.

We employ PPO with a categorical policy head to ensure stable optimization over the discrete camera adjustment actions. PPO maximizes a clipped surrogate objective to prevent destructively large policy updates [30]:

(10) $$L^{CLIP}\left(\theta \right)={\hat{E}}_{t}\left[\min(\rho_{t}\left(\theta \right){\hat{A}}_{t},\mathrm{clip}\left(\rho_{t}\left(\theta \right),1-\epsilon ,1+\epsilon \right){\hat{A}}_{t})\right]$$

where $\rho_{t}\left(\theta \right)=\pi_{\theta }\left(a_{t}|s_{t}\right)/\pi_{\theta_{\mathrm{old}}}\left(a_{t}|s_{t}\right)$ is the probability ratio. As summarized in Figure 3, the state vector $s_{t}=\left[p_{t},r_{t},c_{t},f_{t}\right]$ is jointly fed into the actor and critic branches, while the TE-YOLO confidence is transformed into the threshold-aware reward defined above. The actor outputs one discrete camera adjustment action from A={stay,±x,±y,±z,±θ,±ϕ}, and the critic evaluates the long-term utility of the current observation state.

The resulting information flow is summarized in Figure 3. Unlike a conventional detector-only pipeline, the TE-YOLO outputs are retained as intermediate control signals: the confidence value contributes to reward construction, while the compact feature vector is included in the policy state. The actor and critic therefore operate on perception-aware state information rather than on the camera pose alone.

As illustrated in Figure 3, the proposed controller preserves the standard PPO optimization mechanism but changes how task information enters policy learning. In particular, the thresholded defect response is converted into an explicit reward component, thereby establishing a direct functional connection between TE-YOLO perception and camera viewpoint optimization.

### 3.3. End-to-End Active Inspection Pipeline

The perception module (TE-YOLO) and the active control module (CDR-PPO) are integrated into a sequential closed-loop pipeline. At each time step, the system first captures a lens image from the current camera pose. TE-YOLO then produces defect predictions, confidence values, and a compact visual representation. These outputs are used to construct the PPO state, after which the policy selects one discrete position or orientation adjustment. The adjusted camera pose generates the next observation, from which the confidence-driven reward of the executed action is computed, and closes the perception–decision–action loop.

**Figure 4:**
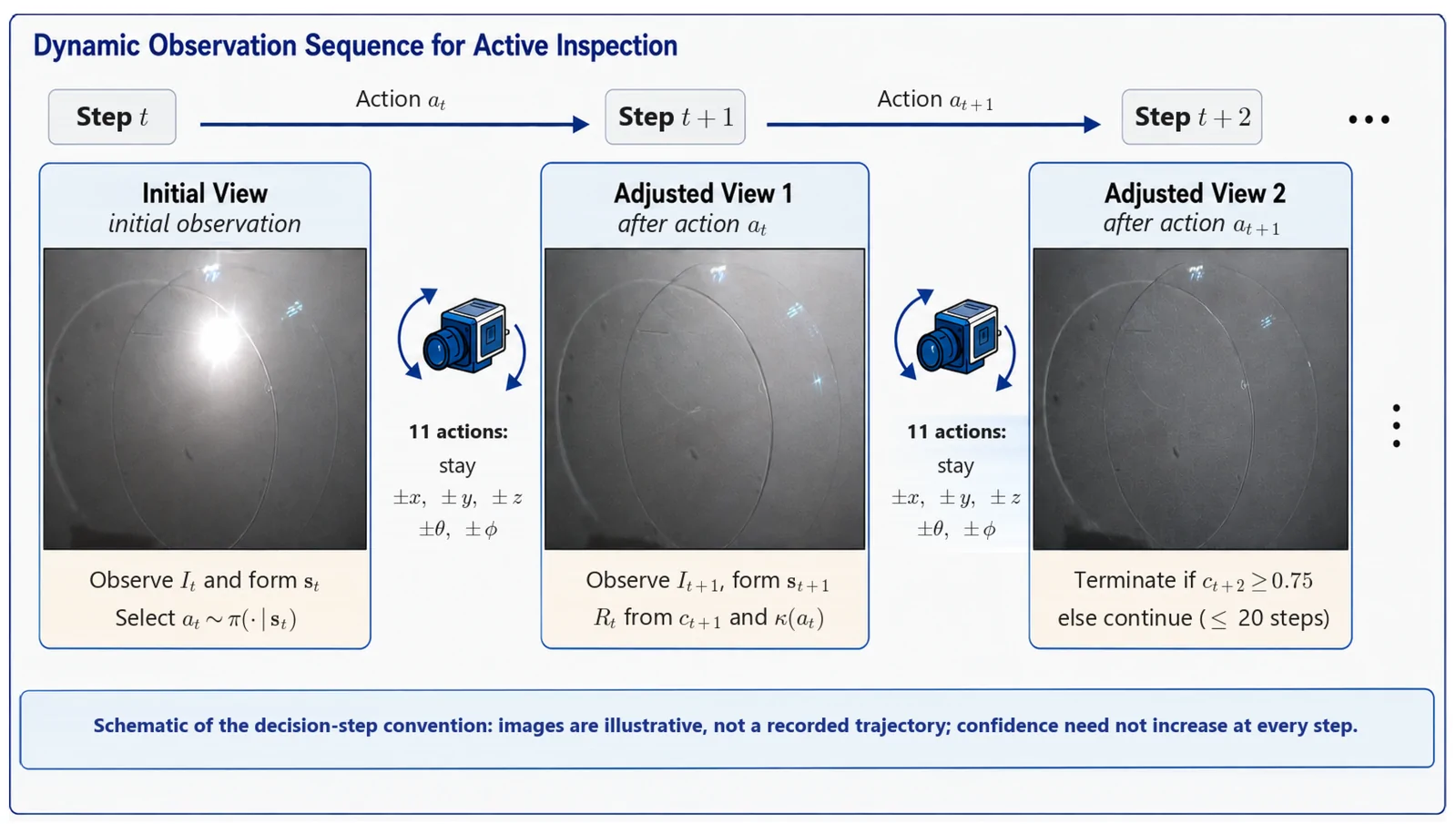
Dynamic observation sequence of the end-to-end active inspection process. The agent starts from an initial glare-contaminated viewpoint and iteratively applies discrete camera adjustments; the reward of each action uses the post-action confidence, and the episode ends when the confidence reaches the threshold or after 20 steps. An ellipsis, where shown, means that the same adjustment cycle continues.

Figure 4 presents the temporal organization of this process. The displayed observations correspond to successive decision intervals rather than to three independent static tests. Consequently, the figure should be interpreted as the partial visualization of a longer iterative sequence that can continue until the confidence criterion is satisfied or the maximum episode length is reached.

The complete computational procedure is summarized in Algorithm 1. The algorithm separates the fixed TE-YOLO inference stage from the trainable PPO update stage while preserving their closed-loop interaction through the confidence-derived reward. Algorithm 1 describes the proposed architecture; in the policy experiments of Section 4, the call of $D_{\omega }$ is replaced by the inspection environment, which returns $c_{t+1}$ and $f_{t+1}$. During each rollout, the current image is converted into perception outputs, the policy selects a camera action, and the resulting transition is stored for subsequent advantage estimation and policy optimization.
**Algorithm 1** End-to-End TE-YOLO and CDR-PPO Active Inspection**Input:** 
Pre-trained TE-YOLO detector $D_{\omega }$, initial PPO policy network $\pi_{\theta }$, value network $V_{\phi }$, maximum steps *T*, batch size *B* episodes**Output:** 
Optimized policy $\pi_{\theta^{*}}$   1:**for** update j=1 to M/B **do**   2:    **for** episode e=1 to *B* **do**   3:        Reset environment; acquire the initial observation at pose $\left(p_{0},r_{0}\right)$ and construct $s_{0}=\left[p_{0},r_{0},c_{0},f_{0}\right]$   4:        **for** time step t=0 to T−1 **do**   5:            Sample discrete camera adjustment $a_{t}∼\pi_{\theta }\left(a_{t}|s_{t}\right)$   6:            Execute $a_{t}$; acquire the next observation at $\left(p_{t+1},r_{t+1}\right)$ and obtain $c_{t+1}$ and $f_{t+1}$ from $D_{\omega }$; construct $s_{t+1}$   7:            Compute $b_{t+1}=I\left(c_{t+1}\geq \tau_{c}\right)$ and $R_{t}$ from Equation (5)   8:            Set $d_{t}=b_{t+1}$ and $u_{t}=I\left(t+1=T\;\mathrm{and}\;d_{t}=0\right)$   9:            Store $\left(s_{t},a_{t},R_{t},s_{t+1},d_{t},u_{t}\right)$ in the rollout buffer 10:            **if** $d_{t}=1$ or $u_{t}=1$ **then** 11:                Exit the time step loop 12:            **end if** 13:        **end for** 14:        Compute advantages ${\hat{A}}_{t}$ and targets ${\hat{G}}_{t}$ with GAE (Equations (7) and (8)) 15:    **end for** 16:    Normalize the advantages over the *B* episodes of the batch 17:    **for** epoch =1 to 10 **do** 18:        For each minibatch of 64 transitions, update $\pi_{\theta }$ by maximizing $L^{CLIP}\left(\theta \right)$ and $V_{\phi }$ by minimizing the mean squared error (MSE) against ${\hat{G}}_{t}$ 19:    **end for** 20:**end for**

## 4. Experiments and Results

### 4.1. Physical Apparatus and Active Vision Evaluation Setup

**Figure 5:**
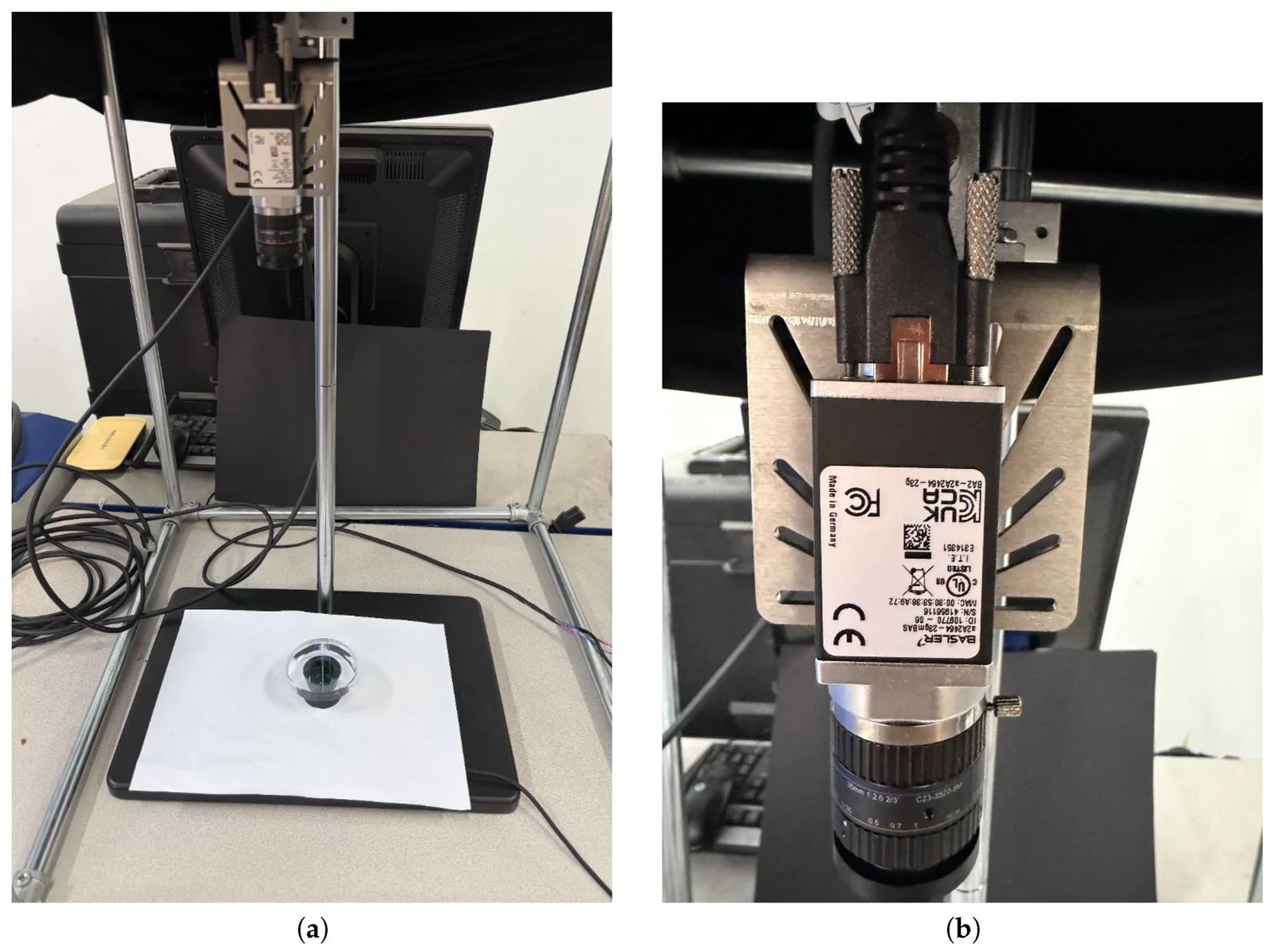
Lens-inspection apparatus. (**a**) Overall arrangement: the camera is mounted on an adjustable support above the lens, which rests on a white sheet on the planar area light source, with a black panel behind the specimen. (**b**) Basler a2A2464-23gmBAS camera with a 35 mm, f/2.0 lens.

The lens images are acquired with the inspection apparatus shown in Figure 5. Images are recorded with a Basler ace 2 a2A2464-23gmBAS monochrome GigE industrial camera fitted with a 35 mm, f/2.0, 2/3-inch fixed-focal-length lens (C23-3520-5M), at 1920 × 1080 pixels. The camera is mounted on an adjustable support above the lens, which rests on a white sheet placed on a planar area light source; a black panel behind the specimen and a black cover above the frame limit stray light. The light source is interchangeable with point and ring sources. During an inspection, the lens, the light source, and the receiving screen are fixed, and only the camera pose is adjusted.

**Figure 6:**
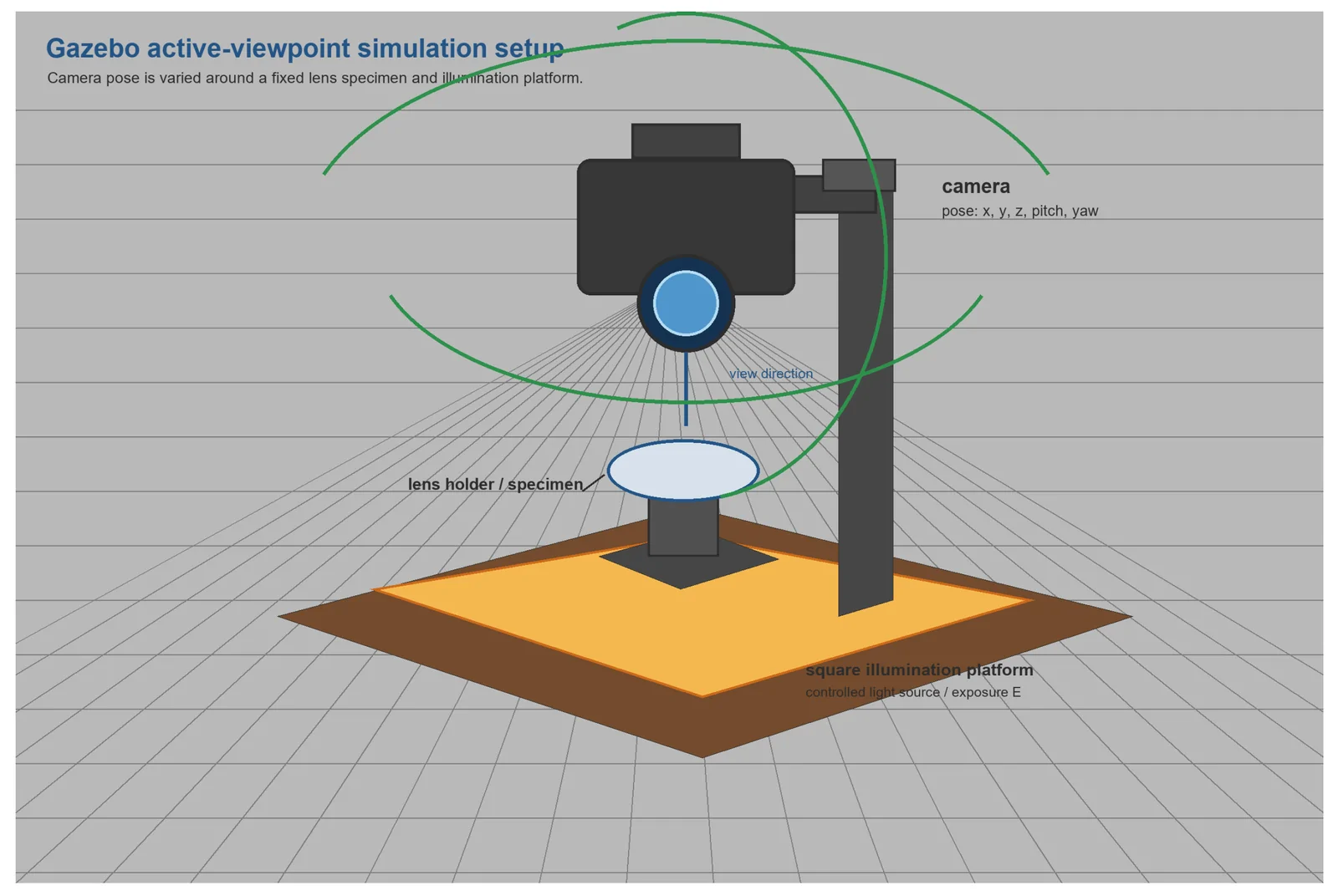
Gazebo scene of the inspection geometry. The scene contains the camera, support column, square illumination platform, lens holder, and camera pose orbit that defines the admissible camera poses of the inspection environment.

On the apparatus, changing the camera configuration moves the specular glare relative to the defect; Section 4.5 shows this in physical acquisition sequences. Training a viewpoint policy and comparing it statistically with baseline policies and reward variants, however, requires thousands of episodes in which every policy receives identical scenarios and initial poses under controlled optical conditions; one training run alone comprises up to 40,000 camera movements (2000 episodes of up to 20 steps). The policy is therefore trained in a simulation environment and is designed for subsequent transfer to the apparatus. For this purpose, we constructed an inspection environment from the apparatus (Figure 6). The Gazebo scene reproduces the geometry of the apparatus and defines the admissible camera poses. Policy training and testing use a parameterized environment built on this geometry, which allows paired, repeatable evaluation over thousands of episodes. Each scenario is defined by a defect position, a light direction, a light intensity, and a surface roughness. These parameters determine a preferred camera pose and a pose-dependent glare ratio. The latent defect visibility probability $v_{t}$ decreases with the distance between the current and the preferred pose and with glare. The confidence $c_{t}$ is a logistic function of $v_{t}$, calibrated by temperature scaling on validation scenarios, and the eight features $f_{t}$ comprise the view quality, normalized intensity and roughness, three pose offsets, and two glare gradient components. The latent visibility $v_{t}$ is not observed by the policy and serves as an evaluation quantity that is independent of the reward.

Training, validation, and test scenarios are assigned to disjoint combinations of binned scenario parameters, so that the test scenarios are parameter combinations not seen in training. Reward weights and the discount factor are selected with training seeds 0–4 on validation scenarios. The selected configuration and all comparison policies are then trained with seeds 10–14, and each final policy is frozen and evaluated once on 200 test episodes per seed with deterministic actions. Initial poses are sampled uniformly from ${\left[-0.35,0.35\right]}^{5}$, and all policies receive identical scenarios and initial poses.

### 4.2. Implementation Details

The lens image dataset contains 208 images of 120 physical lenses with 248 annotated defect boxes. Scratches, foreign particles, and bubbles are merged into a single DEFECT class, because the detector localizes defects without classifying their types. The median box area is approximately 0.14% of the image area. Because several images were acquired from the same lens in consecutive shots, all cross-split image pairs were screened with a perceptual hash and a thumbnail correlation, and each group of near-duplicate images was assigned to a single subset with a 7:2:1 target ratio, so that images identified as near-duplicates of each other, such as consecutive shots of the same lens, are never split across subsets. Table 2 reports the original image-level split and this grouped split.

*Detection module (TE-YOLO)*. The network was initialized with Common Objects in Context (COCO) pre-trained YOLOv8 weights to accelerate convergence on our limited-size physical lens dataset. All detectors were trained with an input size of 1280 pixels, the AdamW optimizer (initial learning rate $10^{-3}$, weight decay $5\times 10^{-4}$), batch size 2, a maximum of 200 epochs, and early stopping with patience of 50 epochs on the validation fitness. The checkpoint with the best validation fitness was evaluated once on the test subset.

*Active control module (CDR-PPO)*. The PPO agent was trained for **2000 episodes** per seed, with each episode capped at a maximum horizon of **20 discrete camera adjustment steps**. Each step corresponds to one active observation cycle. Transitions from 16 consecutive episodes form one training batch, which is used for 10 update epochs over minibatches of 64 transitions. The Adam optimizer was used with a learning rate of $3\times 10^{-4}$, a clipping parameter ϵ=0.2, and a generalized advantage estimation (GAE) parameter λ=0.95 to balance variance and bias in advantage calculation. The actor and critic share a two-layer multilayer perceptron with 128 hidden units and tanh activations.

*Statistical protocol*. To support a reliable comparison and account for the inherent stochasticity of deep reinforcement learning, each configuration is trained with **5 randomized runs** with different seeds for both modules. Results are reported as the mean ± sample standard deviation across seeds, and differences are tested with paired *t* tests across seeds. Detectors were trained on an NVIDIA GeForce RTX 5090 or RTX 5090 D GPU using PyTorch 2.11.0.

### 4.3. Quantitative Results and Policy Analysis

Following the evaluation protocol, the reported results indicate distinct trends in perception accuracy and active control efficiency.

#### 4.3.1. Vision Module Validation on Physical Datasets

To validate the visual perception module, TE-YOLO was evaluated against representative detectors on the lens defect dataset. Table 3 compares TE-YOLO with the baseline YOLOv8, the Transformer-based RT-DETR, and a plain C3 variant that differs from TE-YOLO only in the absence of the Transformer encoder in the final neck block.

**Figure 7:**
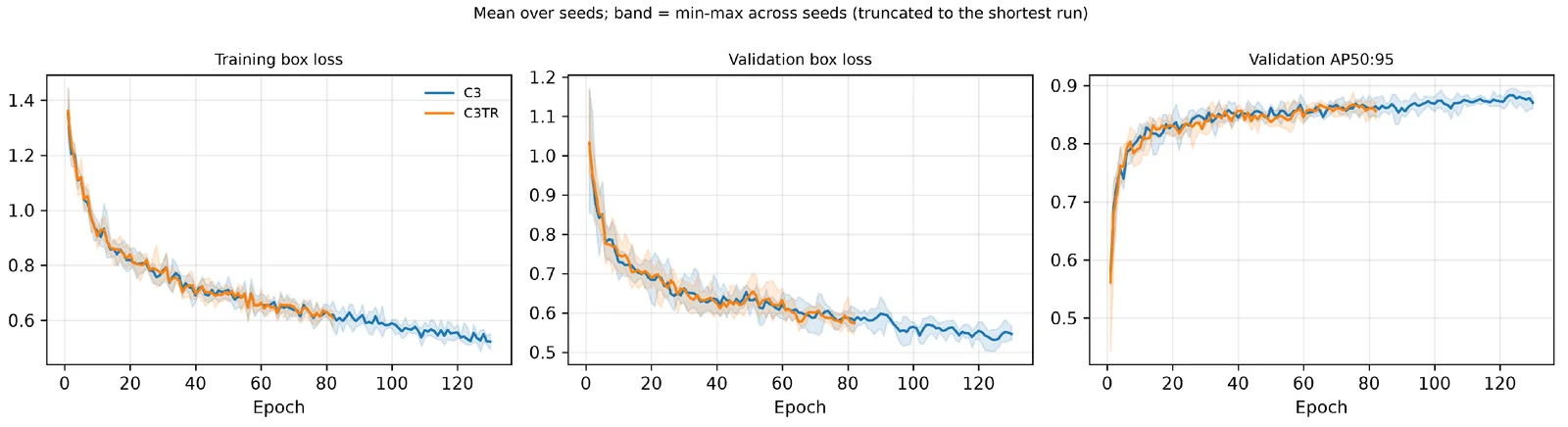
Grouped-split training curves of the plain C3 variant and TE-YOLO: training box loss, validation box loss, and validation AP50:95 per epoch. Lines show the mean over five training seeds and bands show the range across seeds, truncated to the shortest run.

All YOLO-based detectors reach an AP50 close to 0.995 on both splits, so the AP50:95 is the more informative metric. RT-DETR-L has a lower mean and much larger seed-to-seed variance. On the grouped split, this variance arises from one of the five RT-DETR-L runs, which did not converge and was stopped by early stopping at epoch 58; the other four runs reach an AP50:95 between 0.820 and 0.887. TE-YOLO and the plain C3 variant reach the same accuracy: the seed-paired AP50:95 difference is −0.0080±0.0171 on the image-level split (p=0.354) and −0.0063±0.0129 on the grouped split (p=0.339). The C3TR block thus yields no measurable accuracy gain over the plain C3 variant on either split; on both splits, the AP50 is saturated and the test subsets contain 20 and 22 images. Its computational cost is reported in Table 4. Figure 7 shows the training behavior on the grouped split. The validation box loss decreases together with the training loss, and the best validation checkpoint is reached at a median epoch number of 145 for plain C3 and 137 for TE-YOLO.

Table 4 reports the computational cost at the 1280-pixel input. Relative to plain C3, the C3TR block adds 4% of the floating-point operations (FLOPs) and 0.3 ms of latency. Placing it at higher-resolution neck outputs raises the cost steeply because the number of attention tokens grows with the feature map area, and placing it at the backbone end gives the same FLOPs but higher measured latency, so the position before the detection head is the least expensive attention position at this input size. The policy network adds 0.03 ms per decision.

#### 4.3.2. Active Control Policy Evaluation and Ablation

The active control policy was evaluated within the defined active vision environment. For the reward weight selection, $\lambda_{b}\in \left\{1,5,10,20,80\right\}$ and γ∈{0.99,0.95,0.9} were combined with two training schedules (500 episodes with per-episode updates; 2000 episodes with 16-episode batches), giving 30 configurations evaluated on the validation scenarios. Configurations that violated Equation (6) remained at the attainment of the fixed viewpoint (at most 50.7% versus 50.5%). The selected configuration, $\lambda_{b}=10$ and γ=0.9 with batched training, reached 84.7±4.3% on the validation scenarios and is reflected in all results below.

**Figure 8:**
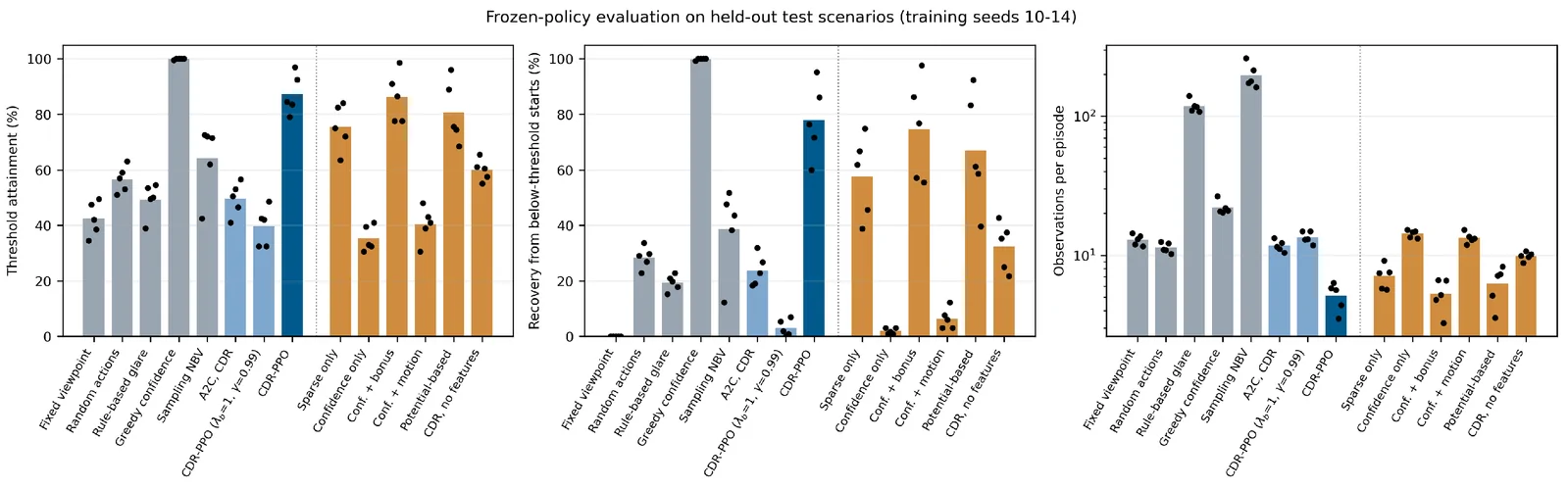
Frozen policy evaluation on held-out test scenarios: threshold attainment, recovery from below-threshold starts, and observations per episode (logarithmic scale). (**Left**) of the dotted line: baselines and CDR-PPO; (**right**): reward and feature ablations. Bars show the mean over training seeds 10–14; dots show individual seeds.

CDR-PPO is compared with a fixed viewpoint, random actions, a rule-based search that moves to the one-step candidate with the lowest glare, a greedy search that moves to the candidate with the highest confidence, a sampling-based next-best-view (NBV) search, advantage actor–critic (A2C) with the same network and reward, and CDR-PPO trained with $\lambda_{b}=1$ and γ=0.99, which violates Equation (6). Search strategies are charged one observation for every evaluated candidate, because each evaluation requires a camera move and an image acquisition. The greedy and rule-based searches evaluate the ten one-step candidates before every move. The NBV search samples 20 candidate poses uniformly within ±2 steps (±0.32) of the current pose in all five coordinates, clipped to the admissible range, and scores each candidate by its calibrated confidence minus 0.05 times its distance to the current pose in units of the step size. If the best score does not exceed the current confidence, it stays; otherwise, it executes one step along the coordinate with the largest difference from the best candidate. A candidate whose confidence exceeds the threshold does not count as attainment. As for the learned policies, attainment is declared only when the image acquired after the executed action reaches the threshold, and this post-action acquisition is charged one further observation. Because the detector confidence enters the reward, we also report the final latent visibility $v_{t}$, which no policy observes. Table 5 and Figure 8 report the results on the held-out test scenarios.

CDR-PPO reaches the threshold in 87.3% of the test episodes with 5.1 observations per episode. It exceeds the fixed viewpoint by 44.9 percentage points (paired 95% interval [32.4,57.4], p<0.001), random actions by 30.7 (p=0.001), the rule-based search by 38.0 (p<0.001), NBV by 23.2 (p=0.026), A2C by 37.8 (p<0.001), and the policy with $\lambda_{b}=1$ and γ=0.99 by 47.7 (p<0.001). The greedy confidence search reaches 99.9% but evaluates all 11 one-step candidates before every move and therefore needs 22.1 observations per episode; CDR-PPO reaches the threshold with about one quarter of these observations, which reduces the number of camera moves and image acquisitions per inspection. The final glare ratio of CDR-PPO (0.181) is lower than that of the fixed viewpoint (0.194; paired difference −0.013, [−0.020,−0.006], p=0.006) and similar to those of greedy search and NBV. The rule-based search reaches the lowest glare (0.101) but only 49.3% attainment, because defect visibility also depends on the distance to the preferred pose.

Because the policies differ strongly in the number of observations that they require, Table 6 compares them at fixed observation budgets. An episode ends at attainment, so it attains within a budget of *K* observations exactly when it reaches the threshold with at most *K* observations. The time per inspection is approximately proportional to the number of observations (Section 5), so a fixed observation budget also corresponds to a fixed inspection time. Among the episodes that start below the threshold, CDR-PPO recovers 68.6% within five and 77.8% within 10 observations. The greedy search recovers none within 10 observations, because its first decision already requires 12 observations (the initial image, ten candidates, and the post-action image); it recovers 32.0% within 22, 76.2% within 34, and 88.8% within 50 observations. Up to a budget of 34 observations per lens, CDR-PPO therefore attains the most episodes of all policies; the greedy search attains more only with larger budgets.

With the same PPO implementation, state, termination rule, and weights, we compare the sparse threshold reward only, confidence only, confidence plus threshold bonus, confidence plus motion cost, the complete CDR reward, and a potential-based variant that combines the threshold bonus with $\gamma c_{t+1}-c_{t}$ and the motion cost [29]. Table 7 reports the results. The two variants without a threshold bonus remain below the fixed viewpoint, and the complete CDR reward exceeds them by 52.0 and 47.0 percentage points (p<0.001). The complete reward attains 11.9 percentage points more than the sparse reward (p=0.053) and reaches an attainment level similar to that of confidence plus bonus (86.2%, p=0.78) and that of potential-based shaping (80.7%, p=0.45). These three variants also contain the threshold bonus, and their differences from the complete reward are not statistically significant at five seeds. The ablation therefore attributes the improvement to the threshold bonus at a weight satisfying Equation (6), rather than to the particular form of the dense term: every variant with the bonus exceeds the fixed viewpoint, and both variants without it remain below. Among the variants with the bonus, the complete reward reaches the threshold in the fewest steps (4.12, compared with 6.11 for the sparse reward and 5.27 for potential-based shaping). Removing the feature vector $f_{t}$ from the state lowers attainment to 59.9±4.0% (difference 27.4 percentage points, [15.8,39.0], p=0.003), so the policy relies on the feature input.

### 4.4. Qualitative Illustration: Detector Behavior Under Glare

**Figure 9:**
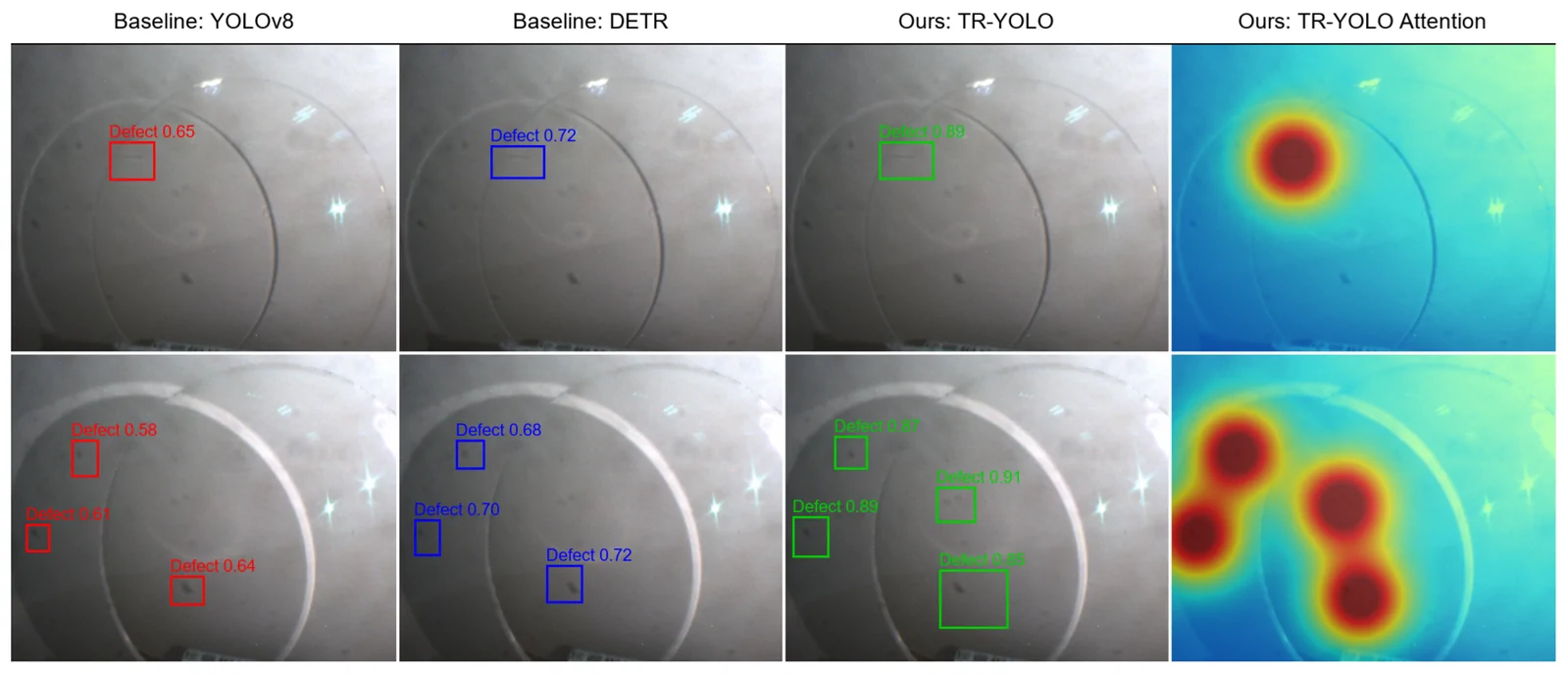
Schematic illustration of the behavior of YOLOv8, DETR, and TE-YOLO on two glare-affected lens images (scratch and foreign particle). Column 4 illustrates the intended focus of TE-YOLO on the defect regions. This figure is placed at its first mention. Colors in column 4 are a schematic heat map of that focus and are not a quantitative scale.

To complement the quantitative analysis of TE-YOLO, Figure 9 illustrates the typical behavior of YOLOv8, DETR, and TE-YOLO on two glare-affected lens images. The figure is a schematic illustration of the behaviors discussed below; the quantitative comparison of the detectors is given in Table 3.

The baseline **YOLOv8** relies on local convolutional receptive fields, so the high-contrast gradients of glare boundaries can resemble defect contours and lead to false positives or reduced confidence near glare.

**DETR** uses global self-attention, which can suppress such responses. Because it lacks the spatial inductive bias of convolutional detectors, its bounding boxes for micro-defects (e.g., dust) tend to be looser than those of YOLOv8.

**TE-YOLO** is designed to combine both properties: it retains the CNN backbone for shallow feature extraction and spatial priors and places the C3TR module before the detection head to add global context. Column 4 of Figure 9 illustrates the intended focus of TE-YOLO on the defect regions in the form of a heat map. Gradient-based activation maps such as Grad-CAM [32] indicate the image regions that contribute most strongly to an individual prediction; in manufacturing-oriented deep learning, they serve as local diagnostics of model focus for individual predictions or process stages [33]. The heat map in Figure 9 is an illustration of this focus and is not used as evidence of an internal attention mechanism.

### 4.5. Qualitative Results: Active Inspection Matrix

**Figure 10:**
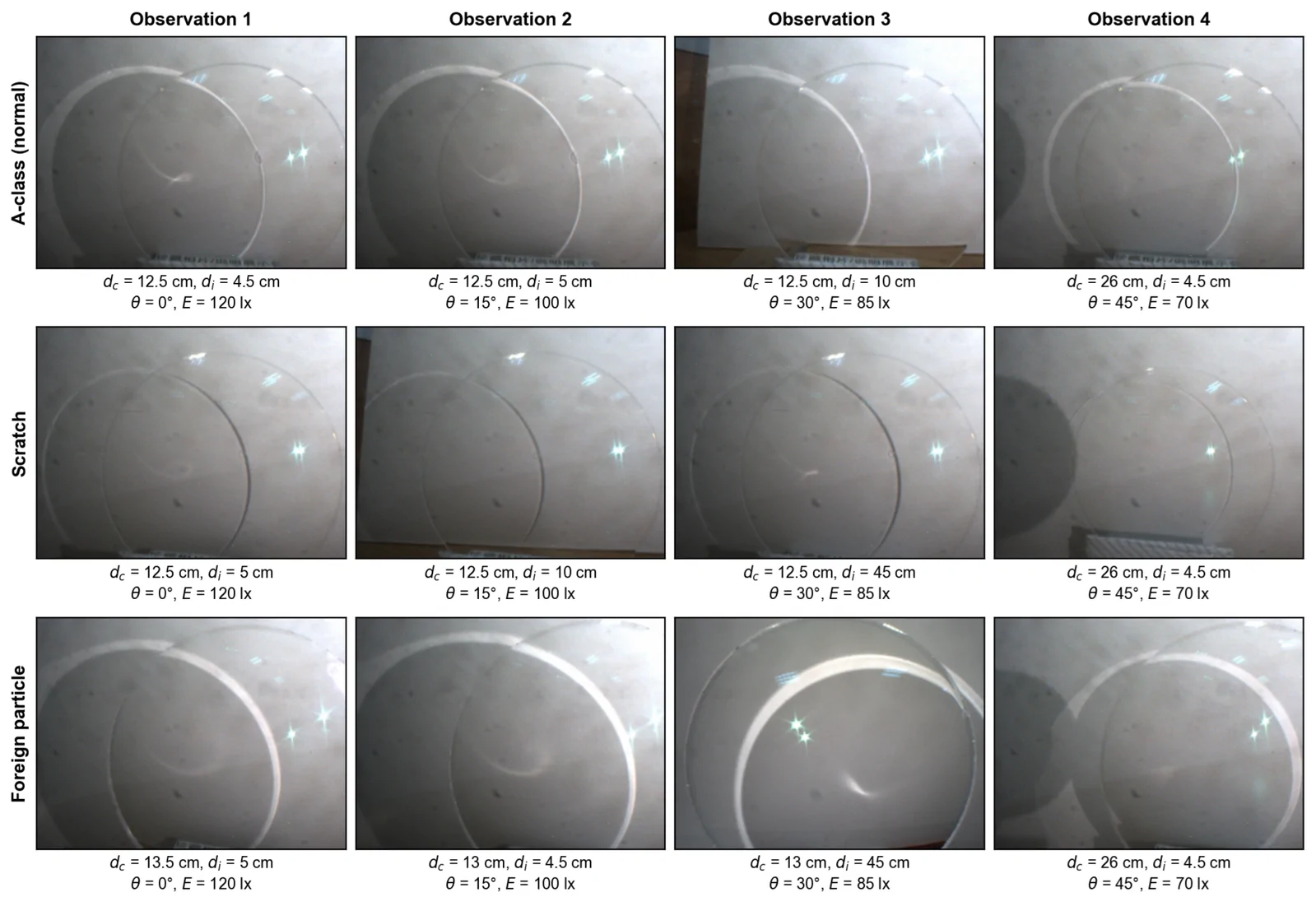
Qualitative evaluation matrix of the active inspection process. Rows represent different lens conditions (A-Class Normal, Scratch, and Foreign Particle). Columns show successive observations of a physical acquisition sequence; the acquisition configuration is given below each observation.

To demonstrate the active vision effect on the physical apparatus, we present a 3×4 qualitative matrix (Figure 10). This matrix shows physical acquisition sequences on the apparatus of Figure 5 for three lens categories (Normal A-Class, Scratch, and Foreign Particle) over four successive observations.

Between successive observations, the acquisition configuration recorded below each image (object distance $d_{c}$, image distance $d_{i}$, pitch angle θ, and illuminance *E*) is changed. At the first observation, specular reflections and the bright edge ring overlap the central region of the lens; as the configuration changes, the reflections and the ring move across the lens, and regions covered in one observation are uncovered in another.

The physical sequences show the effect on which the framework is built: the glare pattern depends on the camera configuration, so a defect masked by glare in one observation can be observed without it in another.

## 5. Discussion

*Inspection time*. At the 1280-pixel input, the TE-YOLO forward pass takes 4.84 ms per image and the policy network takes 0.03 ms per decision (Table 4), so the inspection time is dominated by camera motion and image acquisition. The time per inspection is approximately the number of steps multiplied by the sum of the motion, settling, and acquisition times, and the step interval can be chosen to match the production takt. Reducing the number of observations per inspection is therefore a primary design objective; CDR-PPO needs 5.1 observations per episode, compared with 22.1 for greedy search and 198.3 for NBV (Table 5). At a fixed budget of 10 observations per lens, CDR-PPO recovers 77.8% of the below-threshold starts and the greedy search recovers none (Table 6).

*Detector confidence as the reward signal*. The detector confidence enters both the reward and the threshold criterion, so threshold attainment measures whether the policy reaches a high-confidence observation. For this reason, the policies are additionally evaluated against the latent visibility $v_{t}$, which is independent of the reward. CDR-PPO ends at visibility of 0.794, compared with 0.707 for the fixed viewpoint and 0.811 for greedy search, so the higher confidence that it reaches corresponds to more visible defects.

*Physical and environment-based evidence*. The detector is trained and tested on physical lens images; the physical acquisition sequences on the apparatus show the effect of camera configuration changes on glare; and the statistical comparison of policies is carried out in the inspection environment, where a paired and repeatable evaluation is possible. In manufacturing research, conclusions drawn from data-driven models are strengthened when they are corroborated by an independent physical or physics-based representation, as in the coupling of explainable machine learning with multiphysical simulation in mask electrolyte jet machining [34]. In the present framework, the physical acquisition sequences corroborate the dependence of glare on the camera configuration that the environment represents, and the weight condition of Equation (6) depends only on the threshold and the discount factor, so that it applies directly to the confidence measured on the apparatus. The next step retrains the policy on a state restricted to quantities observable on the apparatus (Section 3), runs it with actual TE-YOLO outputs on the apparatus, and evaluates policy-driven acquisition sequences on more lenses, including normal lenses in the detector training data, with trajectory-level true positive detection, localization intersection over union (IoU), false positives on verified normal lenses, and the inspection time measured against the production takt.

## 6. Conclusions

This paper presents a confidence-driven end-to-end active inspection framework for online quality control in a computer-integrated manufacturing context. The framework is organized around three coupled components: the TE-YOLO perception module, the CDR-PPO viewpoint controller, and a time-sequential dynamic observation process. TE-YOLO places a C3TR module before the detection head to combine local defect representation with global context information. CDR-PPO does not alter the fundamental PPO optimization rule; instead, its contribution lies in a task-specific reward that transforms the detector confidence and its thresholded response into a signal for policy training, together with a camera motion term. The dynamic observation protocol closes the loop by repeatedly acquiring an image, evaluating defects, selecting a discrete position or orientation adjustment, and capturing the next observation. On 208 physical lens images, TE-YOLO reaches an AP50 of 0.995 and an AP50:95 of 0.865 at a small additional cost relative to a plain C3 variant of equal accuracy. Physical acquisition sequences on the apparatus show that changing the camera configuration shifts the glare on the lens. In the inspection environment that reproduces the apparatus geometry, CDR-PPO reaches the confidence threshold in 87.3% of held-out test episodes with 5.1 observations per episode, exceeding the fixed, random, rule-based, NBV, and A2C baselines and requiring about one quarter of the observations of a greedy confidence search; the reward ablation identifies the threshold bonus, weighted according to the derived condition, as its decisive component. Future work will transfer the trained policy to the physical apparatus, evaluate it on more lenses with an independent ground truth on defective and normal lenses, measure the inspection time against the production takt time, and extend the evaluation to additional lens types, manufacturing batches, production-line interfaces, and inspection configurations.

## Figures and Tables

**Table 1:** State, action, reward, and termination definitions used by CDR-PPO. TE-YOLO denotes the Transformer-enhanced YOLO detector.

Component	Symbol	Definition
Camera position	$p_{t}$	Cartesian camera position ${\left[x_{t},y_{t},z_{t}\right]}^{T}$.
Camera orientation	$r_{t}$	Pitch and yaw angles ${\left[\theta_{t},\phi_{t}\right]}^{T}$.
Defect confidence	$c_{t}$	Confidence of the highest-confidence defect prediction; generated by the inspection environment and temperature-calibrated in the policy experiments.
Glare ratio	$g_{t}$	Fraction of the lens region affected by glare, computed by the inspection environment; evaluation metric only.
Visual feature	$f_{t}$	Compact feature vector from TE-YOLO in the proposed architecture; eight environment-generated features in the policy experiments.
Action	$a_{t}$	One of 11 discrete actions: stay, or one step of 0.16 in one pose coordinate.
Threshold attainment	–	$c_{t+1}\geq \tau_{c}$ after the action, with $\tau_{c}=0.75$.
Termination	–	Threshold attainment, or truncation after 20 steps.

**Table 2:** Composition of the lens image dataset.

Split	Training	Validation	Test	Total
Image-level split: images	145	41	22	208
Image-level split: defect boxes	170	47	31	248
Grouped split: images	146	42	20	208
Grouped split: defect boxes	179	47	22	248

**Table 3:** Test results over five training seeds (mean±sample standard deviation). AP50 is the average precision at an intersection-over-union threshold of 0.50, and AP50:95 is the average over thresholds from 0.50 to 0.95.

Split	Model	AP50	AP50:95	Precision	Recall	Params (M)
Image-level	YOLOv8n	0.9950±0.0000	0.8194±0.0127	0.9986±0.0009	0.9992±0.0018	3.011
	RT-DETR-L	0.9590±0.0558	0.7846±0.0701	0.9408±0.0963	0.9806±0.0434	32.808
	Plain C3	0.9950±0.0000	0.8220±0.0078	0.9984±0.0009	1.0000±0.0000	2.842
	TE-YOLO	0.9950±0.0000	0.8140±0.0111	0.9980±0.0000	1.0000±0.0000	2.842
Grouped	YOLOv8n	0.9950±0.0000	0.8760±0.0067	0.9966±0.0012	1.0000±0.0000	3.011
	RT-DETR-L	0.8415±0.3432	0.7247±0.3162	0.8180±0.3940	0.9273±0.1626	32.808
	Plain C3	0.9950±0.0000	0.8711±0.0087	0.9974±0.0004	1.0000±0.0000	2.842
	TE-YOLO	0.9950±0.0000	0.8649±0.0118	0.9972±0.0003	1.0000±0.0000	2.842

**Table 4:** Computational cost at a 1280-pixel input (batch 1, fp32, forward pass; mean±standard deviation over 300 timed passes on one NVIDIA GeForce RTX 5090 D). The policy network is timed on the CPU. GFLOPs denotes $10^{9}$ floating-point operations.

Model	Params (M)	GFLOPs	Latency (ms)	Peak Memory (MB)
Plain C3	2.847	32.2	4.54±0.24	107
TE-YOLO (C3TR at P5 neck)	2.847	33.6	4.84±0.20	177
YOLOv8n	3.011	32.8	4.49±0.22	151
RT-DETR-L	32.808	426.8	19.17±0.42	503
C3TR at P3 neck output	3.001	131.8	67.86±1.50	20,137
C3TR at P4 neck output	2.970	43.7	9.51±1.42	1380
C3TR at backbone end	2.847	33.6	8.56±1.96	173
CDR-PPO policy network (CPU)	0.020	–	0.032±0.015	–

**Table 5:** Frozen policy comparison on held-out test scenarios (mean±sample standard deviation over training seeds 10–14, 200 test episodes per seed). Recovery is attainment among episodes that start below the threshold. Observations (Obs.) include the initial image and, for search strategies, every evaluated candidate pose.

Policy	Attain. (%)	Recovery (%)	Steps	Obs.	Final $v_{t}$	Final $g_{t}$
Fixed viewpoint	42.40±6.21	0.00±0.00	11.94±1.18	12.9±1.2	0.707±0.006	0.194±0.004
Random actions	56.60±4.77	28.40±3.95	10.34±0.96	11.3±1.0	0.635±0.010	0.198±0.004
Rule-based glare search	49.30±6.15	19.33±2.89	10.71±1.17	118.8±12.8	0.561±0.027	0.101±0.005
Greedy confidence search	99.90±0.22	99.85±0.34	1.91±0.23	22.1±2.6	0.811±0.007	0.179±0.003
Sampling-based NBV	64.10±12.83	38.67±15.61	9.39±1.95	198.3±41.0	0.776±0.006	0.184±0.005
A2C, CDR reward	49.50±5.99	23.75±5.65	10.71±1.12	11.7±1.1	0.639±0.058	0.203±0.009
CDR-PPO, $\lambda_{b}=1$, γ=0.99	39.60±6.97	3.00±3.00	12.49±1.32	13.5±1.3	0.547±0.095	0.195±0.004
CDR-PPO	87.30±7.29	77.83±13.49	4.12±1.16	5.1±1.2	0.794±0.013	0.181±0.002

**Table 6:** Threshold attainment (%) within a fixed observation budget *K* on held-out test scenarios (mean±sample standard deviation over training seeds 10–14). Observations are counted as in Table 5. Italic lines are group labels, not policies.

Policy	K=5	K=10	K=22	K=34	K=50
*All test episodes*					
Fixed viewpoint	42.4±6.2	42.4±6.2	42.4±6.2	42.4±6.2	42.4±6.2
Random actions	47.2±5.9	51.0±4.8	56.6±4.8	56.6±4.8	56.6±4.8
Rule-based glare search	0.0±0.0	0.0±0.0	44.4±6.8	48.8±5.8	49.2±6.1
Greedy confidence search	0.0±0.0	0.0±0.0	60.8±5.5	86.2±3.3	93.4±3.8
Sampling-based NBV	0.0±0.0	0.0±0.0	43.7±6.7	43.7±6.7	45.3±7.7
A2C, CDR reward	49.1±5.7	49.5±6.0	49.5±6.0	49.5±6.0	49.5±6.0
CDR-PPO, $\lambda_{b}=1$, γ=0.99	39.6±7.0	39.6±7.0	39.6±7.0	39.6±7.0	39.6±7.0
CDR-PPO	82.0±4.7	87.3±7.3	87.3±7.3	87.3±7.3	87.3±7.3
*Episodes starting below the threshold*					
Fixed viewpoint	0.0±0.0	0.0±0.0	0.0±0.0	0.0±0.0	0.0±0.0
Random actions	14.0±4.0	19.7±3.8	28.4±4.0	28.4±4.0	28.4±4.0
Rule-based glare search	0.0±0.0	0.0±0.0	10.9±2.9	18.4±2.8	19.1±2.8
Greedy confidence search	0.0±0.0	0.0±0.0	32.0±4.2	76.2±3.6	88.8±5.4
Sampling-based NBV	0.0±0.0	0.0±0.0	2.3±1.2	2.3±1.2	5.3±3.5
A2C, CDR reward	23.0±5.1	23.8±5.7	23.8±5.7	23.8±5.7	23.8±5.7
CDR-PPO, $\lambda_{b}=1$, γ=0.99	3.0±3.0	3.0±3.0	3.0±3.0	3.0±3.0	3.0±3.0
CDR-PPO	68.6±8.7	77.8±13.5	77.8±13.5	77.8±13.5	77.8±13.5

**Table 7:** PPO reward ablation on held-out test scenarios (mean±sample standard deviation over training seeds 10–14). All variants use $\lambda_{b}=10$ and γ=0.9.

Reward	Attainment (%)	Recovery (%)	Steps	Final $v_{t}$
Sparse threshold only	75.40±8.33	57.53±14.96	6.11±1.46	0.776±0.021
Confidence only	35.30±4.64	1.90±1.07	13.29±0.88	0.663±0.011
Confidence + threshold bonus	86.20±9.02	74.63±18.30	4.28±1.40	0.791±0.019
Confidence + motion cost	40.30±6.42	6.37±3.81	12.37±1.21	0.677±0.017
Complete CDR	87.30±7.29	77.83±13.49	4.12±1.16	0.794±0.013
Potential-based shaping	80.70±11.37	66.99±20.99	5.27±1.90	0.782±0.024

## Data Availability

The data that support the findings of this study are not publicly available because they were generated using laboratory equipment and are subject to commercial collaboration, third-party confidentiality, and contractual restrictions. Access requests may be directed to the corresponding author and will be considered subject to the applicable institutional and contractual approvals.
